# Development and Validation of a Treatment Algorithm for Osteoarthritis Pain Management in Patients With End-Stage Kidney Disease Undergoing Hemodialysis

**DOI:** 10.1177/20543581241249365

**Published:** 2024-05-13

**Authors:** Mai Mohsen, Jordanne Feldberg, Angelina Abbaticchio, S. Vanita Jassal, Marisa Battistella

**Affiliations:** 1 Leslie Dan Faculty of Pharmacy, University of Toronto, ON, Canada; 2 Department of Nephrology, Toronto General Hospital – University Health Network, ON, Canada; 3 Department of Medicine, University of Toronto, ON, Canada; 4 Department of Pharmacy, University Health Network, Toronto, ON, Canada

**Keywords:** osteoarthritis, hemodialysis, end-stage kidney disease, treatment algorithm, chronic pain management

## Abstract

**Background:**

Although osteoarthritis is common in the hemodialysis population and leads to poor health outcomes, pain management is challenged by the absence of clinical guidance. A treatment algorithm was developed and validated to aid hemodialysis clinicians in managing osteoarthritis pain.

**Objective:**

The objective was to develop and validate a treatment algorithm for managing osteoarthritis pain in patients undergoing hemodialysis.

**Design:**

A validation study was conducted based on Lynn’s method for content validation.

**Setting:**

To develop and validate a treatment algorithm, interviews were conducted virtually by the primary researcher with clinicians from various institutions across the Greater Toronto and Hamilton Area in Ontario.

**Patients:**

The treatment algorithm was developed and validated for the management of osteoarthritis pain in patients on hemodialysis. Patients were not involved in the development or validation of the tool.

**Measurements:**

The algorithm was measured for content and face validity. Content validity was measured by calculating the content validity index of each component (I-CVI) of the algorithm and the overall scale validity index (S-CVI). Face validity was assessed by calculating the percentage of positive responses to the face validity statements.

**Methods:**

A draft algorithm was developed based on literature searches and expert opinion and validated by interviewing nephrology and pain management clinicians. Through consecutive rounds of 1:1 interviews, content and face validity were assessed by asking participants to rate the relevance of each component of the algorithm and indicate their level of agreeability with a series of statements. Following each round, the I-CVI of the algorithm as well as the S-CVI was calculated and the percentage of positive responses to the statements was determined. The research team revised the algorithm in response to the findings. The final algorithm provides a stepwise approach to the non-pharmacologic and pharmacologic management of pain, including topical, oral, and opioid use.

**Results:**

A total of 18 clinicians from 7 institutions across the Greater Toronto and Hamilton Area were interviewed (10 pharmacists, 5 nurse practitioners, and 3 physicians). The average S-CVI of the algorithm across all 3 rounds was 0.93. At least 78% of participants provided positive responses to the face validity statements.

**Limitations:**

An algorithm was developed based on input from clinicians working in the province of Ontario, limiting the generalizability of the algorithm across provinces. In addition, the algorithm did not include the perspectives of primary care providers or patients/caregivers.

**Conclusions:**

An algorithm for the management of osteoarthritis pain in the hemodialysis population was developed and validated through expert review to standardize practices and encourage clinicians to use evidence-based treatments and address the psychosocial symptoms of pain. As the algorithm possesses a high degree of content and face validity, it may improve osteoarthritis pain management among patients undergoing hemodialysis. Future research will assess the implementation of the algorithm across hemodialysis settings.

## Abrégé

**Contexte:**

Bien que l’arthrose soit fréquente et qu’elle entraîne de mauvais résultats de santé chez les patients en hémodialyse, la gestion de la douleur liée à l’arthrose est limitée par l’absence de directives cliniques. Un algorithme de traitement a été développé et validé afin d’aider les cliniciens en hémodialyse à gérer la douleur liée à l’arthrose chez leurs patients.

**Objectifs:**

Développer et valider un algorithme de traitement pour la prise en charge de la douleur liée à l’arthrose chez les patients en hémodialyse.

**Conception:**

Étude de validation menée en utilisant la méthode de Lynn pour la validation du contenu.

**Cadre:**

Pour élaborer et valider l’algorithme, le chercheur principal a mené des entrevues en mode virtuel avec des cliniciens de divers établissements de Hamilton et de la région du Grand Toronto (Ontario).

**Sujets:**

L’algorithme de traitement a été développé et validé pour la prise en charge de la douleur liée à l’arthrose chez les patients en hémodialyse. Ces derniers n’ont pas participé au développement ou à la validation de l’outil.

**Mesures:**

La validité du contenu et la validité apparente de l’algorithme ont été évaluées. La validité du contenu a été mesurée en calculant l’indice de validité de chaque composante (I-CVI) de l’algorithme, ainsi que l’indice de validité à l’échelle globale (S-CVI). La validité apparente a été évaluée en calculant le pourcentage de réponses positives aux énoncés de validité apparente.

**Méthodologie:**

Une ébauche de l’algorithme a été développée à partir de recherches de littérature scientifique et d’avis d’experts, puis validée lors d’entretiens avec des cliniciens en néphrologie et en gestion de la douleur. Afin d’évaluer la validité du contenu et la validité apparente de l’algorithme, les cliniciens ont participé à des séries consécutives d’entretiens individuels où ils devaient évaluer la pertinence de chaque composante de l’algorithme et indiquer leur niveau d’accord avec une série d’énoncés. L’indice de validité du contenu de chaque composante (I-CVI) de l’algorithme, l’indice de validité de l’échelle globale (S-CVI) et le pourcentage de réponses positives aux énoncés ont été calculés après chaque tour. L’algorithme a ensuite été révisé par l’équipe de recherche en réponse aux résultats. L’algorithme final fournit une approche par étapes pour la gestion non pharmacologique et pharmacologique (traitement local, oral, opioïdes) de la douleur.

**Résultats:**

En tout, 18 cliniciens provenant de 7 établissements de Hamilton et de la région du Grand Toronto ont été interviewés (10 pharmaciens, 5 infirmières praticiennes et 3 médecins). L’indice S-CVI moyen de l’algorithme pour les trois séries d’entrevues était de 0,93. Au moins 78 % des participants ont fourni des réponses positives aux énoncés de validité apparente.

**Limites:**

L’algorithme a été élaboré à partir des données fournies par des cliniciens travaillant dans la province de l’Ontario, ce qui limite sa généralisabilité dans les autres provinces. Aussi, l’algorithme n’inclut pas le point de vue des prestataires de soins primaires ou des patients/soignants.

**Conclusion:**

Un algorithme de prise en charge de la douleur liée à l’arthrose chez les patients en hémodialyse a été développé et validé par des experts afin de normaliser les pratiques et d’encourager les cliniciens à utiliser des traitements fondés sur les preuves et à traiter les symptômes psychosociaux de la douleur. L’algorithme ayant montré un degré élevé de validité du contenu et de validité apparente, il a ainsi le potentiel d’améliorer la gestion de la douleur liée à l’arthrose chez les patients en hémodialyse. Les recherches à venir évalueront l’application de l’algorithme dans divers contextes d’hémodialyse.

## Introduction

Burden of pain among persons with end-stage kidney disease (ESKD) on hemodialysis is highly prevalent due to multiple factors, including comorbidities and dialysis treatment itself.^1^ Chronic pain related to osteoarthritis, a degenerative joint disease, is a common concern among patients with ESKD undergoing hemodialysis.^2-7^ Osteoarthritis leads to several complications, including functional impairment, sleep disorders, social isolation, disability, and depression, which may amplify the experience of pain and is an independent risk factor of mortality in the hemodialysis population.^8-14^ Despite its negative health effects, osteoarthritis pain is poorly managed across dialysis units.^15-20^ A key culprit in this dilemma is the absence of clinical guidance applicable to persons with ESKD, including those on dialysis.

The biopsychosocial model of pain is a widely recognized conceptual model for chronic pain management, highlighting that biological, psychological, and social factors influence the perception of pain and must each be addressed for optimal pain management.^21^ Existing osteoarthritis treatment guidelines and tools are not generalizable to the hemodialysis population as they do not adequately address the biopsychosocial factors that modulate the experience of pain in this patient group. For example, guidelines from the American College of Rheumatology (ACR),^22^ and the National Institute for Health and Care Excellence (NICE),^23^ as well as osteoarthritis management tools^24^ do not account for the biological component of pain in patients undergoing hemodialysis as they do not consider patients with impaired kidney function. Although kidney dysfunction results in notable pharmacokinetic and pharmacodynamic alterations, generalized guidelines provide little evidence as to which drugs are safe and efficacious and how to dose adjust in the ESKD population. Thus, providers are hesitant to apply these guidelines to the hemodialysis population due to concerns of safety and toxicity. Moreover, pain management tools specified for patients on hemodialysis^25,26^ do not prioritize the psychosocial management of pain, even though depression/anxiety are common symptoms in patients undergoing hemodialysis and amplify the experience of chronic pain.^1^,^27-29^ Thus, limitations of existing guidance have created knowledge gaps in how osteoarthritis pain can be optimally addressed in the setting of hemodialysis as the perspective of clinicians is often limited by insufficient pain management education.^30-34^

The use of clinical decision support (CDS) tools has been linked to improvements in health care across diverse settings and may aid hemodialysis clinicians (nephrologists, nurse practitioners, pharmacists, and nurses) in the management of osteoarthritis pain.^35,36^ “CDS is a term that has been used to define the myriad of ways in which knowledge is represented in health information and/or management systems to assist health care providers and other stakeholders in patient management decisions.”^37^ Clinical decision support tools can be paper-based or computerized and include a variety of resources, such as algorithms, continuums of care, and treatment models.^37^ Specifically, algorithms provide a series of logically organized instructions to guide clinical decision-making.^38^

Although there is no gold standard method for the validation of treatment algorithms, content and face validity can be achieved through expert review to ensure accuracy, comprehensiveness, and usability of the algorithm. Content validity indicates that the algorithm encompasses all relevant items pertaining to the subject area,^39^ and face validity indicates the algorithm fulfills its stated purpose.^40^ Accuracy and comprehensiveness are achieved by incorporating various topics into the algorithm and aligning the content with clinical practice guidelines and key research evidence.^41^ Usability of the algorithm is achieved through the ability for clinicians to use the algorithm with effectiveness, efficiency, and satisfaction to address the biopsychosocial symptoms of osteoarthritis pain among patients undergoing hemodialysis.^42^

To address the need for guidance applicable to the hemodialysis population, the study team developed and validated a paper-based treatment algorithm to guide a multimodal (psychological, physical, and pharmacological) approach to the management of osteoarthritis pain. The intent of the algorithm is to guide osteoarthritis management based on expert opinion and available literature and is not a clinical practice guideline.

## Methods

This is a 2-phase study involving the development and validation of a treatment algorithm based on Lynn’s method of content validation.^39^ The study was approved by the study site’s institutional research ethics board (21-6079).

### Phase 1: Development of the Treatment Algorithm

The algorithm was developed based on existing osteoarthritis pain management literature^24-26^ and expert opinion. First, a literature search using MEDLINE and Embase was conducted to ascertain that no validated CDS tools, including algorithms, guiding the management of osteoarthritis pain in the hemodialysis population existed (See Supplementary File 1). Next, a literature review was conducted to identify relevant osteoarthritis pain management literature. A focus group consisting of a pharmacy resident, a hemodialysis pharmacist, a pain management pharmacist, a physiatrist, a psychologist, and a nephrologist critically appraised osteoarthritis pain management literature for quality through discussion and drafted the initial version of the algorithm. The focus group was developed through discussions between the primary investigator and the pain management pharmacist to determine which clinicians had expertise in either pain or hemodialysis.

The initial version of the algorithm was created according to the “development stage” of Lynn’s method,^39^ which encompassed 3 steps: domain identification, item generation, and instrument formation.

#### Domain identification

Based on clinical experience, the focus group determined that the algorithm should include 3 main domains related to osteoarthritis pain: assessment, management, and monitoring. Domains were then divided into subsections to ensure a comprehensive approach to pain management. Subsections included history/examination, psychosocial screening, cultural responsiveness, individualized goal setting, pain education, psychological, physical and pharmacological management, monitoring tools and parameters, and pharmacological monitoring.

#### Item generation

Items to be included in the algorithm were extracted from the literature and organized by domain. Items were further assessed by the focus group based on their relevance to the management of osteoarthritis pain in the setting of hemodialysis.

#### Instrument formation

The group opted for a sequential layout starting with assessment, followed by management and monitoring. Management strategies were also arranged sequentially from non-pharmacological approaches to pharmacological modalities. Pharmacological agents were listed in order of increasing adverse effect risk starting with low-risk agents (ie, topicals) and progressing to agents with more substantive risks (ie, opioids). Once group consensus was achieved on all aspects of the algorithm, the initial draft was finalized (see Supplementary File 2).

### Phase 2: Validation of the Algorithm

To assess the algorithm for content and face validity, a 2-part questionnaire was formulated and administered to clinicians through consecutive rounds of 1:1 virtual interviews. Interviews were conducted by 2 researchers from the study team, including a pharmacy resident and a pharmacist/graduate student. Participants were prompted to do their ratings in a standardized way by adhering to the scores outlined in the questionnaires (See Supplementary File 3).

#### Recruitment/interview rounds

According to Lynn, each interview round should include a minimum of 5 and a maximum of 10 clinicians to minimize the risk of chance agreement while sampling from an adequate range of disciplines.^39^ Therefore, 6 clinicians were included per interview round. Clinicians were recruited from various institutions across the Greater Toronto and Hamilton Area. English-speaking, licensed healthcare professionals (physicians, pharmacists, and nurse practitioners) with experience in kidney disease and/or pain management were included. Experience was defined as a minimum of 1 year of direct patient care delivery. Of the 30 clinicians who were approached, 18 agreed to participate and 12 declined primarily because they were busy.

#### The interview questionnaire

Part A of the questionnaire was developed based on the “judgment-quantification” stage described by Lynn^39^ and aimed to assess content validity. Specifically, the initial version of the algorithm was divided into 20 individual components (A to T) and clinicians were asked to assess the relevance of each of these components by providing a rating on a 4-point scale. A score of 1 or 2 indicated the component was less relevant and required either a major change or omission from the algorithm, whereas a score of 3 or 4 indicated that the component was relevant and required either minimal or no change.

Part B of the questionnaire aimed to evaluate face validity. Clinicians were asked to rate a series of statements according to their level of agreement using a 5-point Likert scale. A score of 1 corresponded to strong disagreement, whereas a score of 5 corresponded to strong agreement. Scores of 4 or 5 were regarded as positive responses.

In instances where a lower score was provided (1 or 2 in part A, or 1, 2 or 3 in part B), participants were asked to provide comments to justify their responses. A general comment section was included in the questionnaire to capture additional participant feedback.

Following each round of 6 interviews, participant responses (quantitative scores and qualitative comments) were aggregated and used to revise the algorithm. Any component of the algorithm that received a low score (1 or 2 in part A) by at least 2 out of the 6 participants or any statement that received a lower level of agreeableness (1, 2, or 3 in part B) was considered to require modification by the study team. Once the necessary revisions were completed, the amended algorithm was presented to the next round of participants. The entire process was repeated for an additional 2 rounds until algorithm validation was achieved and the final version was accepted by the study team.

#### Statistical analysis

Part A of the questionnaire assessed content validity by calculating the content validity of each item/component of the algorithm (I-CVI), as well as that of the entire algorithm (S-CVI). The content validity index (CVI) measures the proportion of participants who rated an item as valid. In other words, those who provided a rating of 3 or 4 (ie, agree or strongly agree) out of the total number of participants in a given round of interviews. According to Lynn,^39^ in a round of 6 participants, an item is considered valid when at least 5 out of 6 participants provide a rating of 3 or 4 (I-CVI of ≥ 0.80). Conversely, an item is considered invalid when at least 2 out of the 6 participants provide a rating of 1 or 2 (I-CVI ≤ 0.67). The average of all I-CVIs in a single round is the scale content validity index (S-CVI). The overall S-CVI for the algorithm is calculated by averaging S-CVI across the 3 rounds. The S-CVI threshold, which indicates content validity, is 0.8.^43^

Part B of the questionnaire assessed face validity by calculating the percentage of positive responses. Positive responses were statements rated by participants as either 4 or 5 (agree or strongly agree) on the 5-point Likert scale. The minimum percentage of positive responses indicating adequate face validity is 75%.^44,45^

## Results

### Literature Search

The initial search was conducted in 2021 and retrieved 2 MEDLINE and 76 Embase articles. An updated search in June 2023 retrieved 3 MEDLINE and 93 Embase articles. These searches did not identify a validated CDS tool, including algorithms, for the management of osteoarthritis pain in the hemodialysis setting (See Supplementary File 1). A separate literature review was conducted to inform the initial algorithm and revise its components as needed.

### Study Participants

A total of 18 clinicians from 7 different institutions across the Greater Toronto and Hamilton Area participated in the validation phase of the study. Those included 10 pharmacists, 5 nurse practitioners, and 3 physicians. The participant pool included hemodialysis clinicians, as well as pain management experts. The characteristics of study participants are summarized in Table 1. Characteristics of individual study participants in each interview round are shown in Supplementary File 4.

**Table 1.** Algorithm Validation Participant Characteristics (n = 18).

Characteristic	n (%)
Practice setting	
Hospital 1 (study site)	5 (28)
Hospital 2	6 (33)
Hospital 3	3 (17)
Hospital 4	1 (5.5)
Hospital 5	1 (5.5)
Hospital 6	1 (5.5)
Community	1 (5.5)
City of practice	
Toronto	14 (78)
Burlington	1 (5.5)
Hamilton	3 (17)
Profession	
Physician	3 (17)
Nurse practitioner	5 (28)
Pharmacist	10 (55.5)
Practice area	
Nephrology	3 (17)
Ambulatory Hemodialysis	10 (55.5)
Hemodialysis (rehab)	1 (5.5)
Pain management	2 (11)
Palliative medicine	1 (5.5)
Geriatric and musculoskeletal rehabilitation	1 (5.5)

### Content and Face Validation

Content and face validity for the algorithm was achieved following 3 rounds of interviews. The I-CVI of each component of the algorithm per round of validation is shown in Table 2. The S-CVI calculated for each round of validation was 0.94, 0.88, and 0.98 respectively. The average S-CVI of the algorithm calculated after completion of all 3 rounds was 0.93. This value surpassed the minimum acceptable threshold for content validation (0.8), indicating that participants considered the contents of the algorithm as appropriate and relevant for the management of osteoarthritis pain. The component that consistently required revisions across interview rounds related to psychosocial management. The relatively lower S-CVI score (0.88) was obtained in round 2, possibly because physicians were only included in the second round.

**Table 2.** Content Validity Indices of the Algorithm.

Round 1: algorithm component	I-CVI	S-CVI
A. Assessment (*overview)*	1	0.94
B. Management (*overview)*	0.83	
C. Pain education (*overview)*	1	
D. Psychological management *(overview)*	1	
E. Physical management *(overview)*	1	
F. Pharmacological management *(overview)*	1	
G. Pain education *(overview)*	1	
H. Psychiatry referral	0.83	
I. Physical Management *(overview)*	1	
J. Pharmacological Management *(overview)*	0.83	
K. Monitoring *(overview)*	1	
L. History/examination	1	
M. Psychosocial screening	0.67	
N. Cultural responsiveness	0.83	
O. Individualized goal setting	1	
P. Pain education *(detailed)*	1	
Q. Psychological Management *(detailed)*	1	
R. Physical management *(detailed)*	1	
S. Pharmacological management *(detailed)*	1	
T. Monitoring *(detailed)*	1	
Round 2: Algorithm component	I-CVI	S-CVI
A. Assessment (history/examination)	0.83	0.88
B. Assessment (Psychosocial screening)	0.67	
C. Non-pharmacological management (pain education)	0.83	
D. Non-pharmacological management (physical management)	0.83	
E. Non-pharmacological management (psychological management)	1	
F. Pharmacological management (topical agents)	0.83	
G. Pharmacological management (non-opioid oral agents)	1	
H. Pharmacological management (intra-articular corticosteroids)	0.83	
I. Pharmacological management (opioids)	1	
J. Monitoring	1	
Round 3: Algorithm component	I-CVI	S-CVI
A. Assessment (2 parts)	1	0.98
B. Pain assessment/psychosocial screening	0.83	
C. Basic principles of management	1	
D. Non-pharmacological therapy	1	
E. Topical agents	1	
F. Oral non-opioid agents	1	
G. Intra-articular corticosteroids	1	
H. Opioids	1	
I. Monitoring	1	

The level of participant agreeability with the face validity statements exceeded 75% and is shown in Table 3. The statement with the lowest percentage of positive responses related to the standards of practice.

**Table 3.** Agreement with Face Validity of the Algorithm.

Statement	% Participants who agree or strongly agree
The algorithm is clear and understandable	94
The algorithm uses appropriate language and wording	89
The algorithm flows in a logical manner	89
The algorithm is an accurate representation of the standards of practice	78
I would use the algorithm in my own practice	94
I would be confident recommending the use of this algorithm	89

#### Themes that emerged throughout the validation process

##### Algorithm usability

Participants discussed environmental challenges such as time constraints that could hinder algorithm uptake. Participants recommended a “stepped” approach to osteoarthritis management,^46^ a model of care where pain management interventions are organized into a series of “steps” placed on a continuum from lowest to most intensive.^47^ This approach allowed the study team to reduce the amount of text in the algorithm while retaining valuable clinical information.

##### Psychosocial management

Participants did not unanimously recognize the importance of addressing the psychosocial aspect of chronic pain. Several participants perceived this component to be of lower significance compared with other treatment modalities and resisted its incorporation into the algorithm. Some participants expressed reservations around the language used in the initial draft, indicating that a “psychiatry referral” was not necessary for all patients exhibiting positive symptoms and that “nephrology specific psychiatry” was not an equally accessible service across institutions. Amendments to the language were suggested to emphasize the significance of psychosocial management and enhance the generalizability of the algorithm.

##### Pharmacological management

Participants generally agreed on the pharmacological approach outlined by the algorithm and proposed minor changes to improve its content. For example, the addition of acetaminophen and cannabinoids to the list of agents was advised to clarify their role in therapy. Suggestions related to opioid prescribing were provided by pain specialists and included the addition of tips on opioid initiation and tapering, the omission of fentanyl and oxycodone due to concerns of toxicity, and the inclusion of methadone due to its clinical utility in the hemodialysis population. Notably, several participants had minimal experience with buprenorphine and methadone and did not view their use as a “standard of practice” in the hemodialysis setting.

#### Algorithm development

The initial algorithm consisted of several pages. The first outlined a flow diagram with high-level management steps, and subsequent pages detailed individual management steps. Participants perceived this layout as lengthy and impractical, and expressed confusion regarding the use of the different treatment modalities. Therefore, throughout the validation process, the algorithm underwent major structural modifications to enhance its clarity and usability.

A stepped approach was adopted following Bruyère et al’s^46^ layout in an algorithm for managing knee osteoarthritis. Succinctness of the algorithm was achieved by eliminating the first page of the initial draft and combining relevant components as appropriate. For example, “psychosocial screening” was combined with “psychosocial management” and “cultural responsiveness” with “individualized goal setting.” The recommendations on “pain education” and “physical management” were abbreviated and grouped together under “non-pharmacological modalities.” To facilitate a logical approach to “assessment and screening,” this step was divided into 2 broad components. The first guided diagnosis (red flags and osteoarthritis signs) and the second proposed action items following diagnosis (“assess” baseline pain and “screen” for psychosocial symptoms).

In the final version, the algorithm was prefaced with a list of conditions (eg, psoriatic arthritis) to be ruled out prior to the assertion of an osteoarthritis diagnosis. Also, common osteoarthritis signs and symptoms were included to offer guidance with assessment. To prioritize psychosocial management in the overall care plan, “psychosocial screening” was specified as part of the early assessment step. Moreover, “basic principles of management” was added to the algorithm to encourage the combination of treatment modalities. Additional tips on opioid use were included to increase clinicians’ comfort level with narcotic prescribing. The final version of the algorithm following validation and revisions is shown in Supplementary Figures.

## Discussion

A treatment algorithm was developed and validated to aid clinicians in managing osteoarthritis pain. The algorithm was developed to standardize practices, discourage the use of non–evidence-based treatments, prompt clinicians to address the psychosocial symptoms of pain, and continuously re-evaluate therapy. As the algorithm shows a high degree of content and face validity, it may improve the quality of osteoarthritis pain management among patients undergoing hemodialysis.

To the study team’s knowledge, this is the first validated algorithm for osteoarthritis pain management in the hemodialysis population. Similar algorithms and tools have been developed to guide treatment in other areas of kidney disease management such as anemia,^48^ constipation,^49^ uremic pruritus,^38^ hyperphosphatemia,^50^ and mineral bone disorder.^51^ Studies which evaluated the impact of such tools have demonstrated their utility in improving the overall quality of health care. For example, a study that examined the effectiveness of an algorithm for treating pruritus revealed the usefulness of the algorithm in terms of changes in medications.^52^ Another study suggested that applying an anemia management algorithm decreased the total hemoglobin variability compared with a population protocol-based approach.^53^ It is therefore expected that the use of this algorithm would affirm better patient care.

Although the team was determined to address the psychosocial component of pain, as “pain and depression are highly intertwined and may co-exacerbate physical and psychological symptoms”,^54^ several participants expressed resistance to the incorporation of psychosocial management into the algorithm. This finding corroborates considerable research indicating that mental health is frequently overlooked in the setting of hemodialysis. A scoping review that evaluated how mental health care is provided to adults treated with dialysis in Canada found that mental health care is under-addressed and that “clinical pathways for the assessment and management of mental illness or symptoms in individuals treated with dialysis in Canada are also limited.”^55^ Similarly, in a survey of Canadian nephrologists’ views on psychosocial care in hemodialysis units, mental health treatment was viewed as inadequate.^56^ Therefore, to prioritize the psychosocial management of pain, psychosocial screening was incorporated in the early assessment step along with a link to a free mental health resource (Bounce Back Ontario)^57^ for patients requiring mental health services. In the final round of validation, 5 out of 6 participants were amenable to the incorporation of psychosocial management into the algorithm.

Although the algorithm prompts clinicians to prioritize the psychosocial component of pain, psychology and psychiatry services are often not readily available to the hemodialysis population. Other non-pharmacological interventions including physical and occupational therapies are also difficult to access. Barriers to accessing these therapies include long wait-times for publicly funded services and high out-of-pocket costs. The high costs are often beyond the reach of most persons undergoing hemodialysis who frequently experience loss of income. Further research is needed to understand how these ancillary services/therapies can be delivered to support patients with osteoarthritis on hemodialysis.

The algorithm was developed to bridge knowledge gaps in the use of pain medications and introduce clinicians to pharmacological options that are commonly overlooked in the hemodialysis setting. For example, methadone and buprenorphine are opioids often assumed to be prescribed only for addiction despite their utility in chronic pain management.^58^ They were included in the final version of the algorithm as they exhibit minimal accumulation in renal failure and may provide better alternatives to traditional opioids.^58,59^ Methadone has the benefit of reducing the development of opioid tolerance and preventing glutamate excitotoxicity (which is implicated in the pathogenesis of chronic pain)^32,60^ but requires individualized dosing due to its complex pharmacokinetics.^61^ Thus, the algorithm recommends methadone therapy, but prompts clinicians to consult experienced methadone prescribers. Compared with traditional opioids, buprenorphine has an improved safety and side effect profile and equivalent or greater clinical analgesic efficacy.^62-68^ The algorithm advocates the use of buprenorphine by providing dosing guidance for different formulations. Although hemodialysis clinicians may not be comfortable prescribing buprenorphine due to the lack of experience with the drug, the ongoing HOPE Consortium Trial^69^ will provide valuable insights on the use of buprenorphine in patients on dialysis. Results of this trial may encourage hemodialysis clinicians to adopt the use buprenorphine for chronic pain management.

It is notable that many participants emphasized the need to enhance the usability of the algorithm in a busy dialysis environment, as the uptake of CDS tools including algorithms has been historically poor.^70,71^ Existing literature indicates that the effectiveness of CDS tools in clinical settings is strongly influenced by clinicians’ perceptions. For example, a study that evaluated the impact of physician compliance with CDS systems for anemia management in patients undergoing hemodialysis indicated that physician compliance impacted the overall efficacy of the tool.^72^ Similarly, a qualitative systematic review and meta-aggregation of barriers and enablers to implementing and using CDS systems for chronic diseases revealed that CDS tools perceived to present useful clinical knowledge were more likely to be adopted by clinicians, while those perceived as time-consuming and disruptive to workflow were less likely to be accepted.^73^ Therefore, to enhance algorithm usability, a stepped approach to osteoarthritis management was incorporated in the final version.

This study is the first to develop and validate an algorithm for a relatively small population that tends to be overlooked, addressing an important care gap. Strengths of our study include the involvement of a diverse participant pool, varying by clinician type and location of practice, who provided input on the development and validation of the algorithm. Furthermore, the use of Lynn’s method to interpret and organize data based on expert opinion in 3 rounds of validation allowed the research team to implement multiple rounds of feedback into the final algorithm. Several limitations should be noted. First, the literature search may have not captured all relevant CDS tools for the management of osteoarthritis pain in the hemodialysis setting as it was narrowed to the English language. Second, the findings are limited by the lack of a universal measure for validating CDS tools, including algorithms. Although Lynn’s method is recognized as an acceptable tool, there is no complete agreement in the literature on the threshold for content validity or the ideal method for calculating the S-CVI of CDS tools. Third, there was an uneven distribution of clinicians in the study sample and across rounds. Pharmacists represented 55% of the participant pool and physicians represented only 17% and were solely included in the second round of interviews. Furthermore, not all participant comments could be included into the algorithm to enhance succinctness; thus, some relevant clinical comments may have been excluded. However, the validated algorithm achieved a balance between usability and clinical utility. Finally, the study team acknowledges that the exclusion of primary care providers (PCPs) and patients and/or caregivers is a limitation of this work. This study was conducted as part of a residency project during the COVID-19 pandemic. Thus, given the limited timeline that was available to this project and the difficulty with recruiting clinicians, the study team limited participant inclusion to clinicians with experience in pain and/or hemodialysis. Including PCPs, who are often more skilled in mental health and chronic pain issues than practitioners in the dialysis unit, may have highlighted additional recommendations to address pain management. Similarly, including patients on hemodialysis may have highlighted pain management strategies that are important to patients. Future work should include PCP and patient perspectives in the development and validation of osteoarthritis pain management tools to enhance the relevance and applicability of these tools to the hemodialysis population.

## Conclusions

An algorithm for the management of osteoarthritis pain in the hemodialysis population was developed and validated through literature searches and expert review to help address an important care gap. The algorithm achieved a high degree of content and face validity and may improve osteoarthritis pain management among patients undergoing hemodialysis. Future research is needed to assess the implementation of the algorithm across real-world hemodialysis settings.

## Supplemental Material

sj-docx-1-cjk-10.1177_20543581241249365 – Supplemental material for Development and Validation of a Treatment Algorithm for Osteoarthritis Pain Management in Patients With End-Stage Kidney Disease Undergoing Hemodialysis Supplemental material, sj-docx-1-cjk-10.1177_20543581241249365 for Development and Validation of a Treatment Algorithm for Osteoarthritis Pain Management in Patients With End-Stage Kidney Disease Undergoing Hemodialysis by Mai Mohsen, Jordanne Feldberg, Angelina Abbaticchio, S. Vanita Jassal and Marisa Battistella in Canadian Journal of Kidney Health and Disease

sj-jpg-3-cjk-10.1177_20543581241249365 – Supplemental material for Development and Validation of a Treatment Algorithm for Osteoarthritis Pain Management in Patients With End-Stage Kidney Disease Undergoing Hemodialysis Supplemental material, sj-jpg-3-cjk-10.1177_20543581241249365 for Development and Validation of a Treatment Algorithm for Osteoarthritis Pain Management in Patients With End-Stage Kidney Disease Undergoing Hemodialysis by Mai Mohsen, Jordanne Feldberg, Angelina Abbaticchio, S. Vanita Jassal and Marisa Battistella in Canadian Journal of Kidney Health and Disease

sj-jpg-4-cjk-10.1177_20543581241249365 – Supplemental material for Development and Validation of a Treatment Algorithm for Osteoarthritis Pain Management in Patients With End-Stage Kidney Disease Undergoing Hemodialysis Supplemental material, sj-jpg-4-cjk-10.1177_20543581241249365 for Development and Validation of a Treatment Algorithm for Osteoarthritis Pain Management in Patients With End-Stage Kidney Disease Undergoing Hemodialysis by Mai Mohsen, Jordanne Feldberg, Angelina Abbaticchio, S. Vanita Jassal and Marisa Battistella in Canadian Journal of Kidney Health and Disease

sj-jpg-5-cjk-10.1177_20543581241249365 – Supplemental material for Development and Validation of a Treatment Algorithm for Osteoarthritis Pain Management in Patients With End-Stage Kidney Disease Undergoing Hemodialysis Supplemental material, sj-jpg-5-cjk-10.1177_20543581241249365 for Development and Validation of a Treatment Algorithm for Osteoarthritis Pain Management in Patients With End-Stage Kidney Disease Undergoing Hemodialysis by Mai Mohsen, Jordanne Feldberg, Angelina Abbaticchio, S. Vanita Jassal and Marisa Battistella in Canadian Journal of Kidney Health and Disease

sj-jpg-6-cjk-10.1177_20543581241249365 – Supplemental material for Development and Validation of a Treatment Algorithm for Osteoarthritis Pain Management in Patients With End-Stage Kidney Disease Undergoing Hemodialysis Supplemental material, sj-jpg-6-cjk-10.1177_20543581241249365 for Development and Validation of a Treatment Algorithm for Osteoarthritis Pain Management in Patients With End-Stage Kidney Disease Undergoing Hemodialysis by Mai Mohsen, Jordanne Feldberg, Angelina Abbaticchio, S. Vanita Jassal and Marisa Battistella in Canadian Journal of Kidney Health and Disease

sj-jpg-7-cjk-10.1177_20543581241249365 – Supplemental material for Development and Validation of a Treatment Algorithm for Osteoarthritis Pain Management in Patients With End-Stage Kidney Disease Undergoing Hemodialysis Supplemental material, sj-jpg-7-cjk-10.1177_20543581241249365 for Development and Validation of a Treatment Algorithm for Osteoarthritis Pain Management in Patients With End-Stage Kidney Disease Undergoing Hemodialysis by Mai Mohsen, Jordanne Feldberg, Angelina Abbaticchio, S. Vanita Jassal and Marisa Battistella in Canadian Journal of Kidney Health and Disease

sj-jpg-8-cjk-10.1177_20543581241249365 – Supplemental material for Development and Validation of a Treatment Algorithm for Osteoarthritis Pain Management in Patients With End-Stage Kidney Disease Undergoing Hemodialysis Supplemental material, sj-jpg-8-cjk-10.1177_20543581241249365 for Development and Validation of a Treatment Algorithm for Osteoarthritis Pain Management in Patients With End-Stage Kidney Disease Undergoing Hemodialysis by Mai Mohsen, Jordanne Feldberg, Angelina Abbaticchio, S. Vanita Jassal and Marisa Battistella in Canadian Journal of Kidney Health and Disease

sj-jpg-9-cjk-10.1177_20543581241249365 – Supplemental material for Development and Validation of a Treatment Algorithm for Osteoarthritis Pain Management in Patients With End-Stage Kidney Disease Undergoing Hemodialysis Supplemental material, sj-jpg-9-cjk-10.1177_20543581241249365 for Development and Validation of a Treatment Algorithm for Osteoarthritis Pain Management in Patients With End-Stage Kidney Disease Undergoing Hemodialysis by Mai Mohsen, Jordanne Feldberg, Angelina Abbaticchio, S. Vanita Jassal and Marisa Battistella in Canadian Journal of Kidney Health and Disease

sj-pdf-2-cjk-10.1177_20543581241249365 – Supplemental material for Development and Validation of a Treatment Algorithm for Osteoarthritis Pain Management in Patients With End-Stage Kidney Disease Undergoing Hemodialysis Supplemental material, sj-pdf-2-cjk-10.1177_20543581241249365 for Development and Validation of a Treatment Algorithm for Osteoarthritis Pain Management in Patients With End-Stage Kidney Disease Undergoing Hemodialysis by Mai Mohsen, Jordanne Feldberg, Angelina Abbaticchio, S. Vanita Jassal and Marisa Battistella in Canadian Journal of Kidney Health and Disease
