# Supplementary material for: Development and Validation of a Treatment Algorithm for Osteoarthritis Pain Management in Patients With End-Stage Kidney Disease Undergoing Hemodialysis
Source: Can J Kidney Health Dis. 2024 May 13;11:20543581241249365. doi: 10.1177/20543581241249365 (PMC11092542; doi:10.1177/20543581241249365)
Supplement: sj-docx-1-cjk-10.1177_20543581241249365 – Supplemental material for Development and Validation of a Treatment Algorithm for Osteoarthritis Pain Management in Patients With End-Stage Kidney Disease Undergoing Hemodialysis [file sj-docx-1-cjk-10.1177_20543581241249365.docx]

**Supplementary file 1**. Search strategy

Ovid MEDLINE: Epub Ahead of Print, In-Process & Other Non-Indexed Citations, Ovid MEDLINE® Daily and Ovid MEDLINE® <1946-Present>

| **#** | **Query** | **Results from 13 Oct 2021** |
| --- | --- | --- |
| 1 | exp Osteoarthritis/ | 68,920 |
| 2 | limit 1 to english language | 59,140 |
| 3 | exp Renal Dialysis/ | 118,540 |
| 4 | limit 3 to english language | 100,048 |
| 5 | 2 and 4 | 36 |
| 6 | exp Pain/ | 420,553 |
| 7 | 5 and 6 | 2 |

Ovid MEDLINE: Epub Ahead of Print, In-Process & Other Non-Indexed Citations, Ovid MEDLINE® Daily and Ovid MEDLINE® <1946-Present>

| **#** | **Query** | **Results from 12 Jun 2023** |
| --- | --- | --- |
| 1 | exp Osteoarthritis/ | 76,889 |
| 2 | limit 1 to english language | 66,883 |
| 3 | exp Renal Dialysis/ | 125,883 |
| 4 | limit 3 to english language | 107,181 |
| 5 | 2 and 4 | 38 |
| 6 | exp Pain/ | 457,258 |
| 7 | 5 and 6 | 3 |

Embase Classic+Embase <1947 to 2021 October 13>

| **#** | **Query** | **Results from 14 Oct 2021** |
| --- | --- | --- |
| 1 | exp osteoarthritis/ | 150,509 |
| 2 | limit 1 to english language | 129,954 |
| 3 | exp hemodialysis/ | 123,721 |
| 4 | limit 3 to english language | 111,018 |
| 5 | exp pain/ | 1,513,663 |
| 6 | limit 5 to english language | 1,363,422 |
| 7 | 2 and 4 and 6 | 76 |

Embase Classic+Embase <1947 to 2021 October 13>

| **#** | **Query** | **Results from 14 Oct 2021** |
| --- | --- | --- |
| 1 | exp osteoarthritis/ | 150,509 |
| 2 | limit 1 to english language | 129,954 |
| 3 | exp pain/ | 1,513,663 |
| 4 | limit 3 to english language | 1,363,422 |
| 5 | renal dialysis.mp. or exp hemodialysis/ | 125,205 |
| 6 | limit 5 to english language | 112,305 |
| 7 | 2 and 4 and 6 | 76 |

Embase Classic+Embase <1947 to 2023 June 09>

| **#** | **Query** | **Results from 12 Jun 2023** |
| --- | --- | --- |
| 1 | exp osteoarthritis/ | 170,456 |
| 2 | limit 1 to english language | 149,203 |
| 3 | exp pain/ | 1,716,788 |
| 4 | limit 3 to english language | 1,561,312 |
| 5 | renal dialysis.mp. or exp hemodialysis/ | 143,059 |
| 6 | limit 5 to english language | 129,873 |
| 7 | 2 and 4 and 6 | 93 |
