# Supplementary figures and images for: Development and Validation of a Treatment Algorithm for Osteoarthritis Pain Management in Patients With End-Stage Kidney Disease Undergoing Hemodialysis
Source: Can J Kidney Health Dis. 2024 May 13;11:20543581241249365. doi: 10.1177/20543581241249365 (PMC11092542; doi:10.1177/20543581241249365)

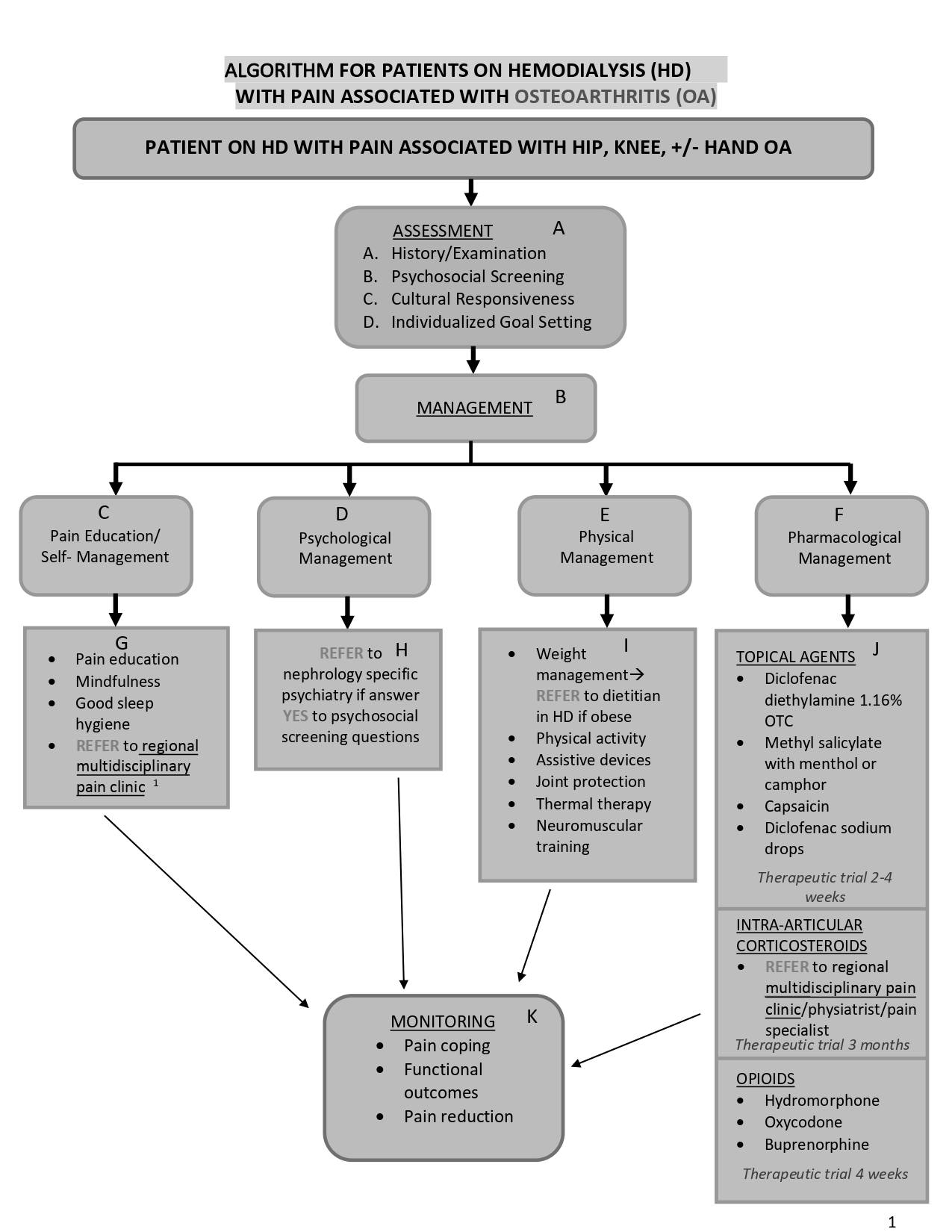

Supplement: sj-jpg-3-cjk-10.1177_20543581241249365 – Supplemental material for Development and Validation of a Treatment Algorithm for Osteoarthritis Pain Management in Patients With End-Stage Kidney Disease Undergoing Hemodialysis [file sj-jpg-3-cjk-10.1177_20543581241249365.jpg]

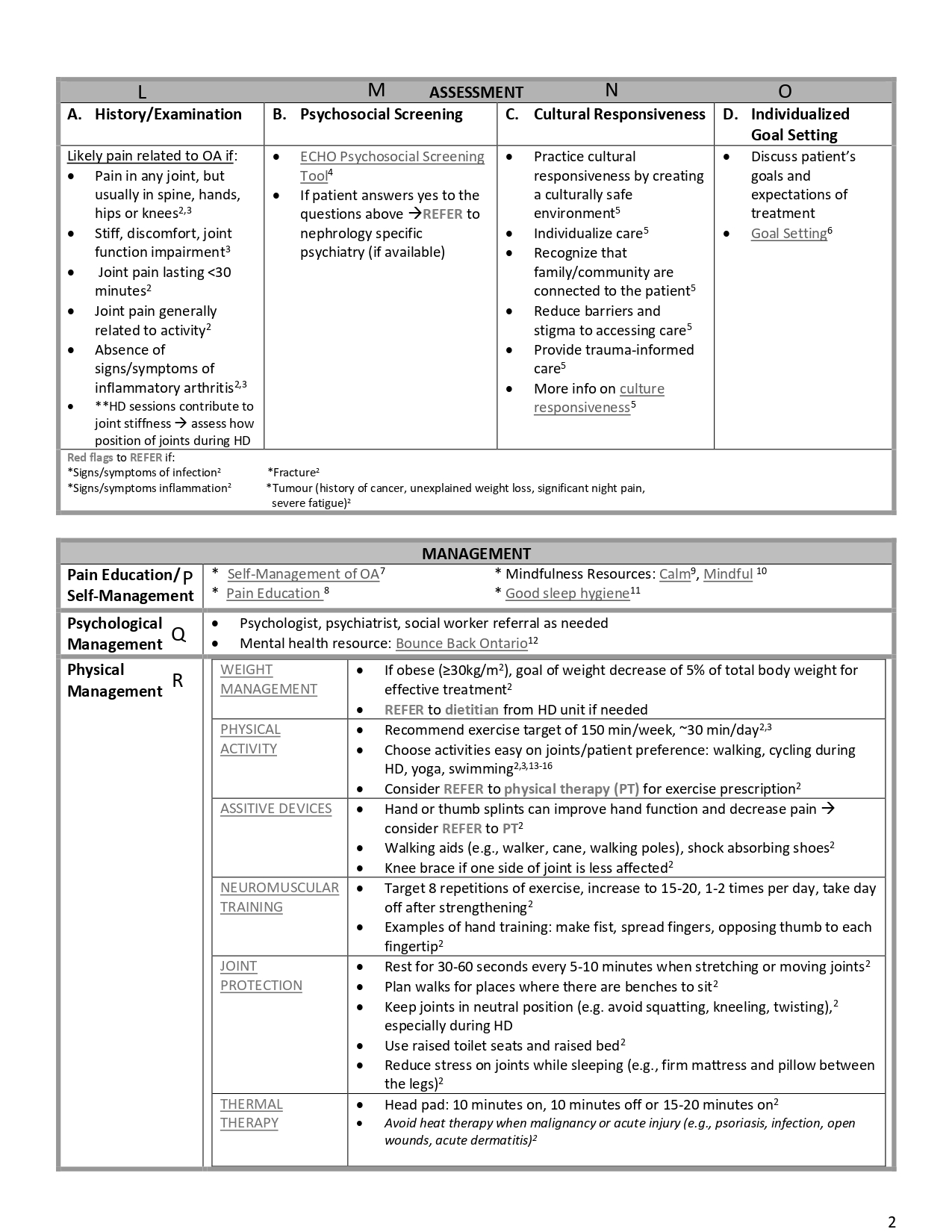

Supplement: sj-jpg-4-cjk-10.1177_20543581241249365 – Supplemental material for Development and Validation of a Treatment Algorithm for Osteoarthritis Pain Management in Patients With End-Stage Kidney Disease Undergoing Hemodialysis [file sj-jpg-4-cjk-10.1177_20543581241249365.jpg]

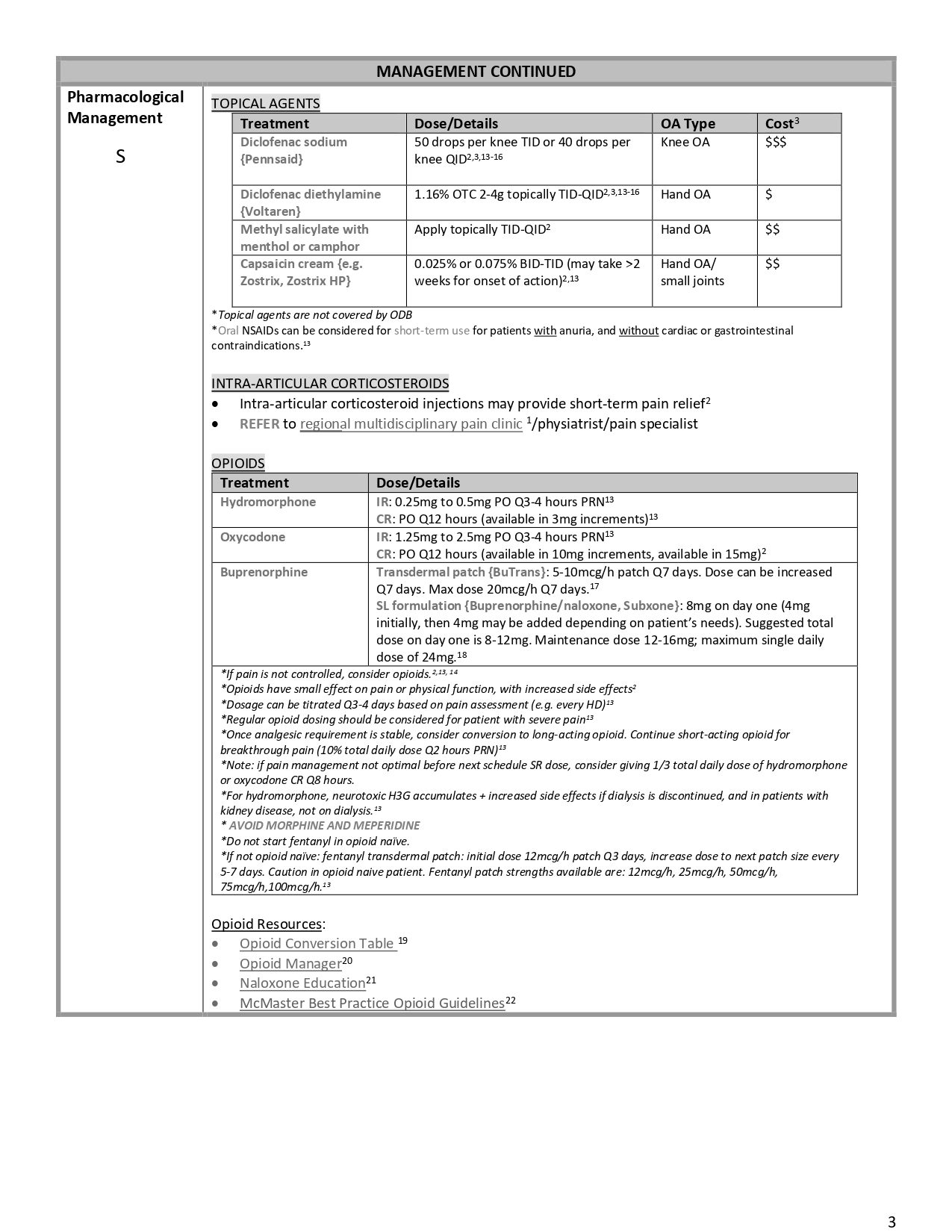

Supplement: sj-jpg-5-cjk-10.1177_20543581241249365 – Supplemental material for Development and Validation of a Treatment Algorithm for Osteoarthritis Pain Management in Patients With End-Stage Kidney Disease Undergoing Hemodialysis [file sj-jpg-5-cjk-10.1177_20543581241249365.jpg]

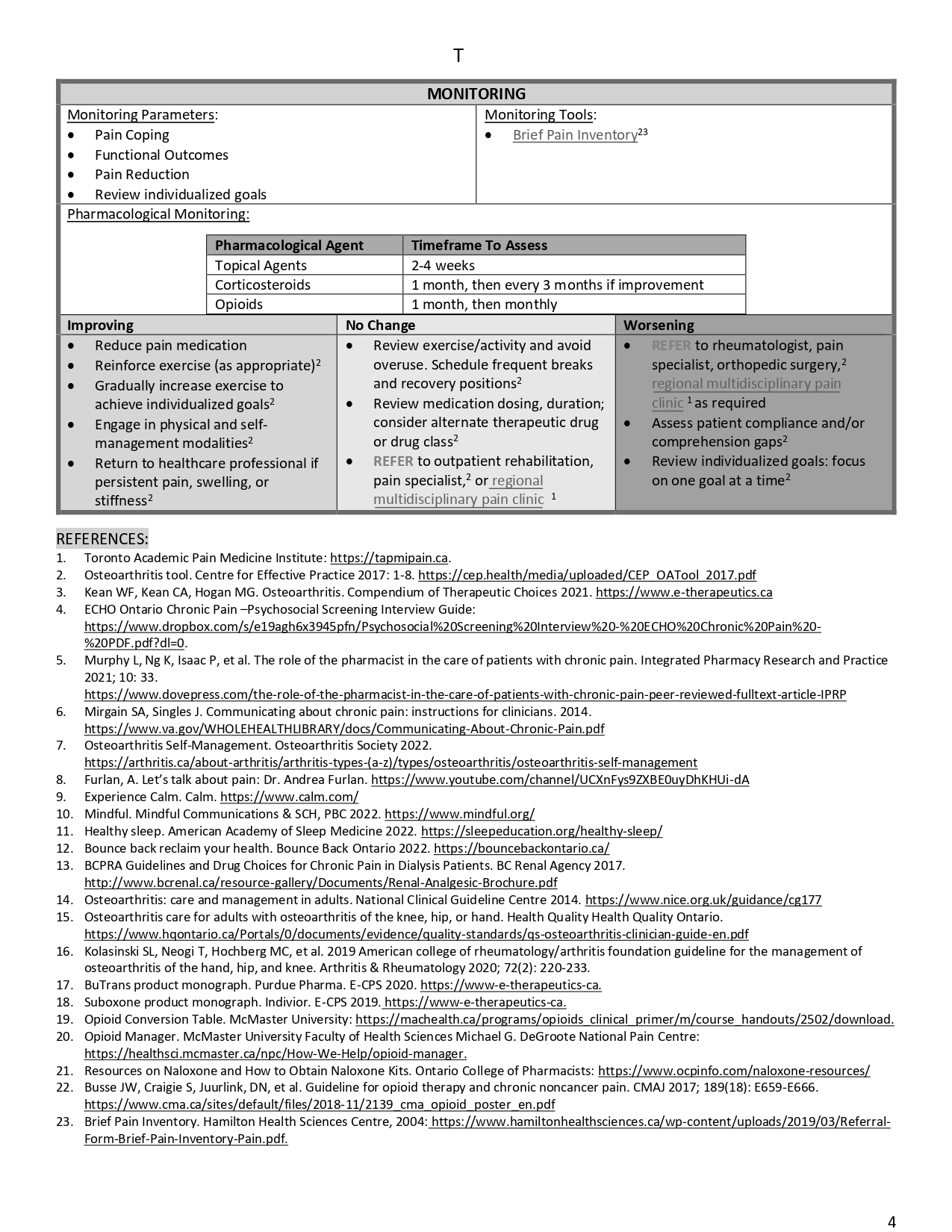

Supplement: sj-jpg-6-cjk-10.1177_20543581241249365 – Supplemental material for Development and Validation of a Treatment Algorithm for Osteoarthritis Pain Management in Patients With End-Stage Kidney Disease Undergoing Hemodialysis [file sj-jpg-6-cjk-10.1177_20543581241249365.jpg]

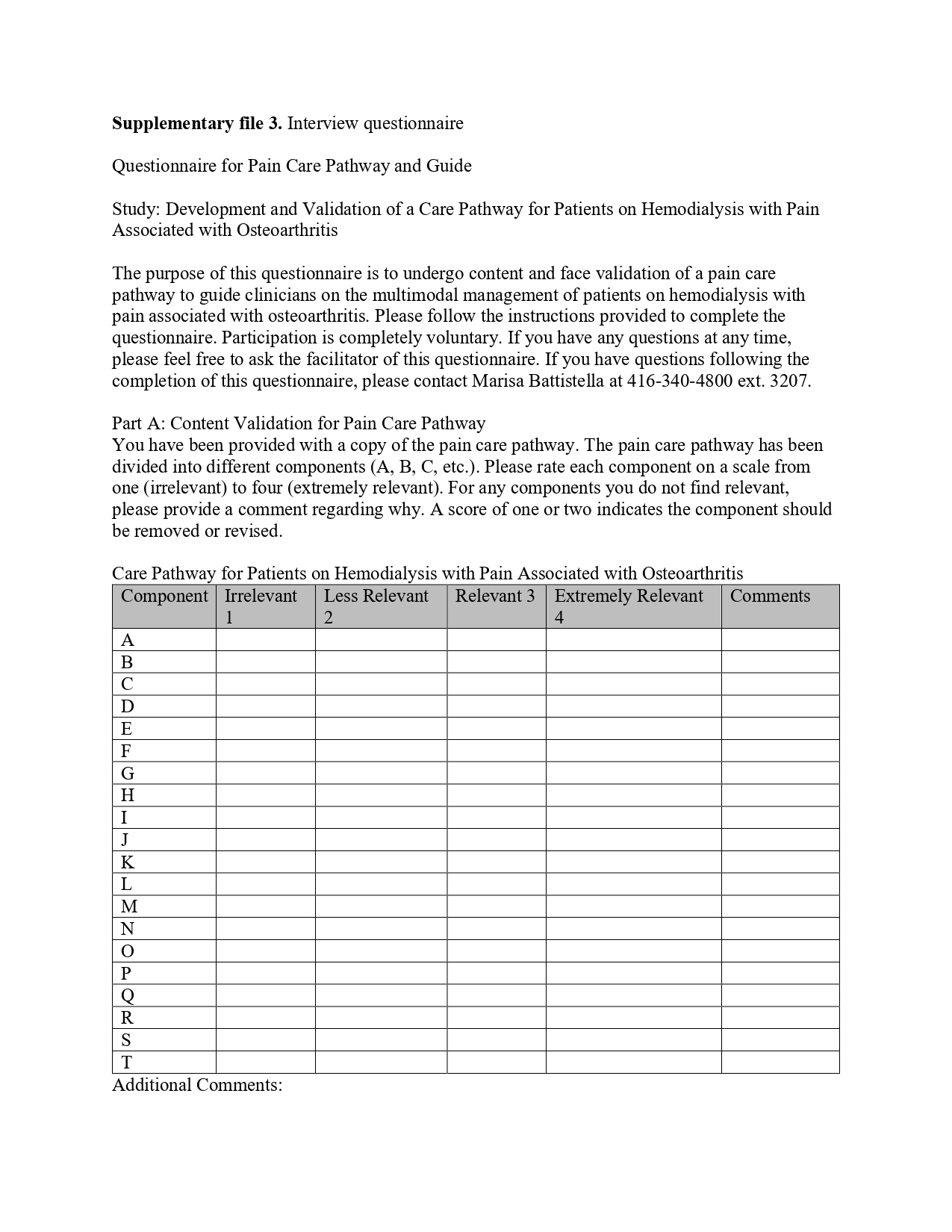

Supplement: sj-jpg-7-cjk-10.1177_20543581241249365 – Supplemental material for Development and Validation of a Treatment Algorithm for Osteoarthritis Pain Management in Patients With End-Stage Kidney Disease Undergoing Hemodialysis [file sj-jpg-7-cjk-10.1177_20543581241249365.jpg]

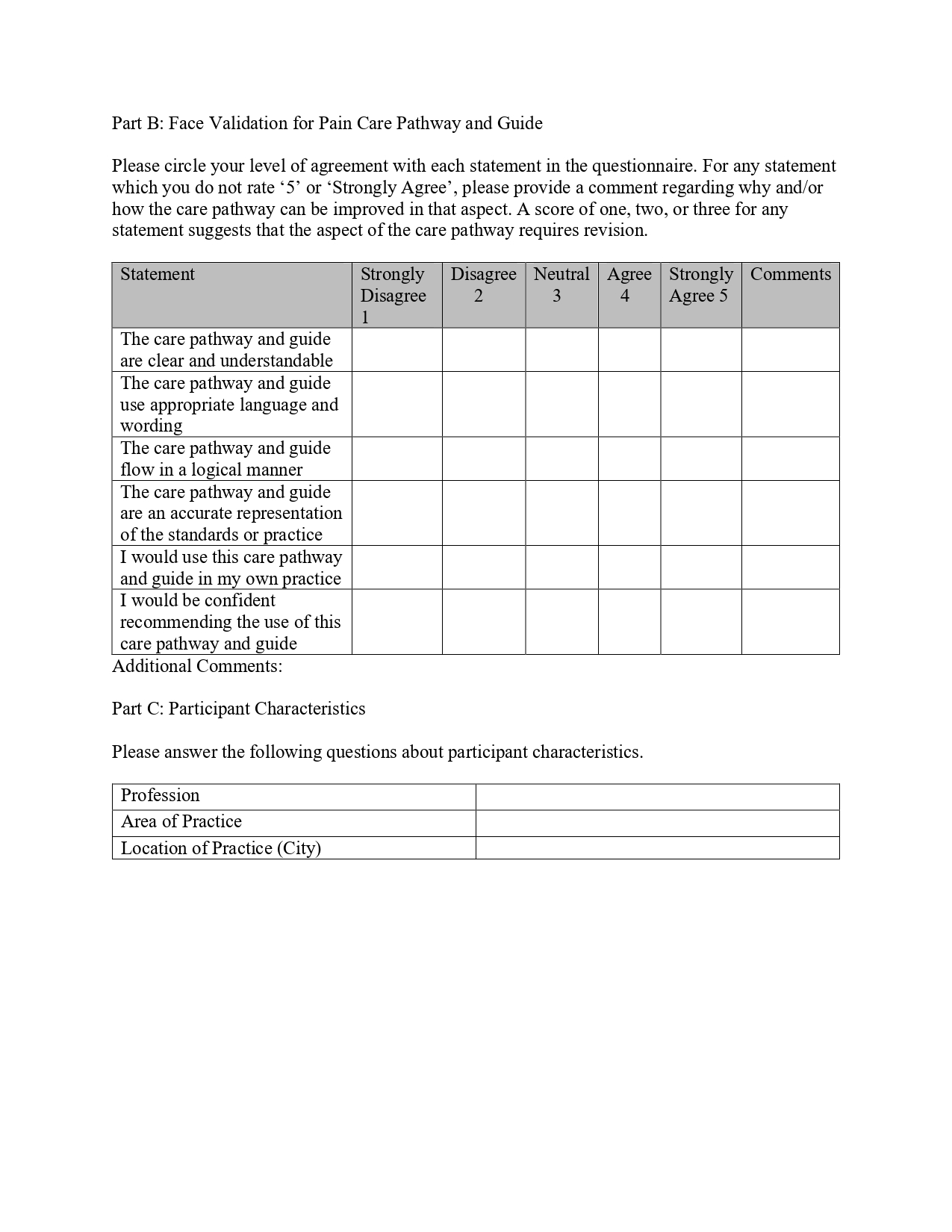

Supplement: sj-jpg-8-cjk-10.1177_20543581241249365 – Supplemental material for Development and Validation of a Treatment Algorithm for Osteoarthritis Pain Management in Patients With End-Stage Kidney Disease Undergoing Hemodialysis [file sj-jpg-8-cjk-10.1177_20543581241249365.jpg]

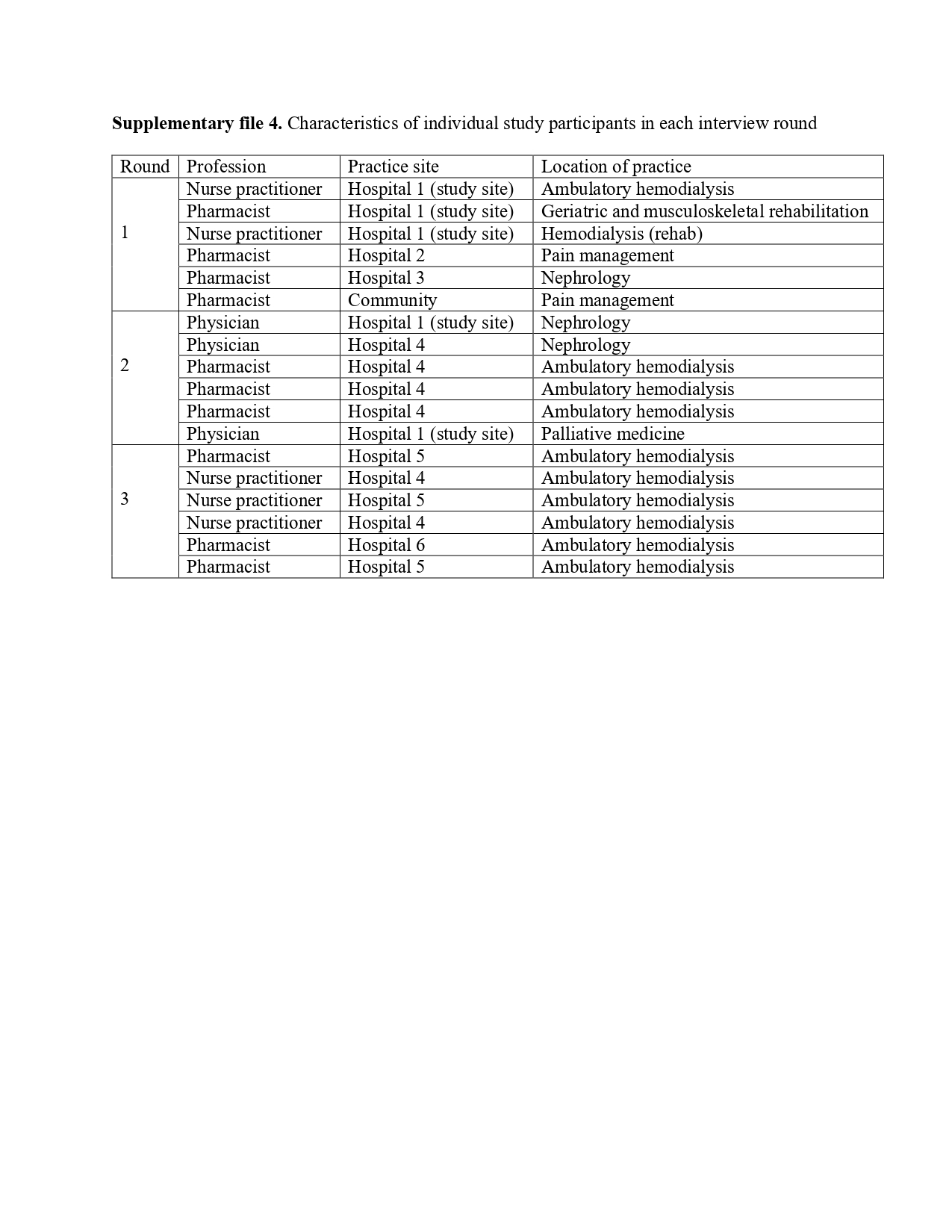

Supplement: sj-jpg-9-cjk-10.1177_20543581241249365 – Supplemental material for Development and Validation of a Treatment Algorithm for Osteoarthritis Pain Management in Patients With End-Stage Kidney Disease Undergoing Hemodialysis [file sj-jpg-9-cjk-10.1177_20543581241249365.jpg]
