# Supplementary material for: Development and Validation of a Treatment Algorithm for Osteoarthritis Pain Management in Patients With End-Stage Kidney Disease Undergoing Hemodialysis
Source: Can J Kidney Health Dis. 2024 May 13;11:20543581241249365. doi: 10.1177/20543581241249365 (PMC11092542; doi:10.1177/20543581241249365)
Supplement: sj-pdf-2-cjk-10.1177_20543581241249365 – Supplemental material for Development and Validation of a Treatment Algorithm for Osteoarthritis Pain Management in Patients With End-Stage Kidney Disease Undergoing Hemodialysis [file sj-pdf-2-cjk-10.1177_20543581241249365.pdf]

Algorithm for patients on haemodialysis (HD) with pain associated with osteoarthritis (OA)

Assessment and screening

This pathway is not intended for use in the following cases:
Inflammatory arthritis, Septic arthritis, Monoarticular arthritis, Infection, Inflammation, Hip/knee replacement, Previous fracture, Crystal arthropathies, Tumour (history of cancer, unexplained weight loss, significant night pain, severe fatigue).[1,2]

Likely pain related to OA if: [1]
- Pain in any joint, but usually in knees, hips, or hands
- Characterised by stiffness, discomfort, joint function impairment
- Joint pain lasting < 30 minutes upon waking
- Joint pain generally related to end of day pain/activity/overuse

- **Assess baseline pain and functional interference.** Click here for the Brief Pain Inventory [3]
- **Screen for psychosocial symptoms of pain.**[4] Click here for screening tool (ECHO)[5]
  - If patient screens + for psychosocial symptoms ***REFER*** to social worker, psychologist, or nephrology dedicated psychiatry as needed.
  - Click here for free mental health resource (Bounce Back Ontario)[6]
    Skill building program delivered via phone with a coach and/or via online videos.

Basic principles of management

Combination of treatment modalities including nonpharmacological and pharmacological therapies is strongly recommended. Set SMART [7] goals and individualize therapy. Provide a culturally safe environment.[8]

Step 1: Non-pharmacological therapy

- **Protective modalities**: Assess position of joints during HD (keep joints in a neutral position), reduce stress on joints during sleep (firm mattress, pillow between legs)[1], use raised beds and toilet seats[1], rest for 30-60 sec every 5-10 min when stretching or moving joints [1]
- **Physical management**: Heat therapy, assistive devices, neuromuscular training, physical exercise, weight management [1]
  REFER to PT/OT as needed. ***REFER*** to dietician/weight loss clinic if obese (BMI≥30kg/m^2)
- **Patient education/ information**: Self-management[9,10], meditation[11,12], sleep hygiene[13]

Step 2: Add a Topical agent if patient is symptomatic and pain is localized

Not covered under ODB, cost may be a barrier

*Preferred **Diclofenac diethylamine (Voltaren emulgel®, Voltaren emulgel ES®) $**	**Methyl salicylate with menthol/ camphor $$**	**Capsaicin cream (Zostrix®, Zostrix HP®) $$**	**Topical CBD/THC $$$**
• 1.16%, 2.32% (2-4 g topically TID-QID)[1,14]<br>• May cause bruising<br>**Diclofenac sodium (Pennsaid®) $$$**<br>• Knee OA 40 drops/knee QID [1] or 50 drops/knee TID	• Hand OA (Topically TID-QID)[1]	• Hand OA/small joints 0.025% or 0.075% BID-QID[1,14]<br>• May take> 2 weeks for onset of action, adherence can be a problem	• Lack of evidence to support its use.[15]<br>• May ***REFER*** to a cannabis clinic for a trial

Combine step 2 and 3 for severe pain

Monitor therapy in 2-4 weeks
scroll down to monitoring

Step 3: Add an oral agent if patient still symptomatic

NSAIDS
- Short term use may be considered for patients with anuria and without cardiac or gastrointestinal contraindications [2]
- Consider gastric ulcer risk [2]

Acetaminophen
- Published evidence has not demonstrated clinically relevant effects on pain/function [16-18]
- Associated with liver dysfunction and hypertension[19]
- May cause INR fluctuations at doses ≥ 2g/day for several days [20]

Monitor therapy in 2 weeks
scroll down to monitoring

Step 4: Trial an intra-articular corticosteroid if patient still symptomatic

- Intra-articular corticosteroid injections may provide short-term pain relief for Hip/Knee OA [1]
- May be administered by MRP or ***REFER*** to rheumatologist, physiatrist, pain specialist or regional multidisciplinary pain clinic [7]

Monitor therapy in 2 weeks, then in 3 months
scroll down to monitoring

Step 5: Add a short-term opioid course to enable non-pharmacological modalities

Opioid management tips

- Do not initiate opioids without an exit strategy. Withdrawal can occur following days, weeks, or months of therapy. May consider tapering by reducing the dose by 5 to 10% of original dose every 2 to 4 weeks.[21,22]
- Ensure there is a single opioid prescriber on the patient's care team who assumes responsibility for adjusting the opioid regimen.
- Titrate slowly based on pain assessment but emphasize improvement in function as a primary goal.
- Opioids have a small effect on chronic pain/physical function with increased side effects (constipation, respiratory depression, fractures, hyperalgesia)[23]. Continue therapy only if there is clinically meaningful improvement in pain and function[24] e.g., 30% improvement from baseline on a 3-item PEG scale [25,26]
- Consider a prophylactic bowel regimen to manage constipation (senna glycosides +/-PEG+/- lactulose).[2]
- Co-prescribe naloxone for at-risk patients (high opioid dosages ≥ 50MME/day, concurrent benzodiazepine use, history of overdose, history of substance use disorder, during tapers or for patients residing with children who are at risk of accidental poisoning.[27]
- **Opioid resources: Naloxone education [28], Opioid conversion table[29], Opioid manager[30], Opioid guideline for chronic non-cancer pain [31]**

Avoid Morphine and Meperidine [14]

Buprenorphine

- Favourable side effect profile (less risk of respiratory depression, tolerance, hyperalgesia, minimal renal elimination)[32]
- Special considerations for managing acute/postoperative pain in patients receiving buprenorphine therapy [33]
- If patient is switching from an opioid, ***REFER*** to pain specialist for buprenorphine induction[34]
- If patient is **not** on opioids, may initiate buprenorphine as follows:

SL formulation buprenorphine/naloxone (Suboxone®) [35-37]
2mg initially, then up titrated depending on patient's needs.
Maximum suggested daily dose of 16-24mg.
Pain control may be optimized with BID dosing.

♦ General benefit under ODB (available in 2/0.5mg, 8/2mg)

Transdermal patch (Butrans®) [38]
5-10mcg/h patch Q7 days. Dose can be **increased Q7 days**. Max dose 20 mcg/h Q7 days.[14]

♦ Not a benefit under ODB (available in 5,10,15,20 mcg/h patches)**$$$**

Hydromorphone

- Neurotoxic H3G accumulates if dialysis is discontinued [14,39,40]
- **IR** preferred to minimize the risk of accumulation.

Initial IR dosing: 0.5mg to 1mg PO Q3-4 hours PRN [2,14]
Extending the dosing interval is recommended [40] (e.g., Q6-8 h)

♦ General benefit under ODB

CR dosing: PO Q12 hours [14]

♦ General benefit under ODB (3mg, 4.5mg, 6mg, 9mg, 12mg, 18mg)
♦ Require EAP (24mg & 30mg)

Methadone

- It is of clinical value in the HD population.
- It demonstrates a complex pharmacokinetic profile [41]
- ***REFER*** to an experienced methadone prescriber (pain specialist, chronic pain clinic or palliative care)

♦ Only 10mg/ml oral liquid concentrate is covered by ODB

Monitor therapy in 2-4 weeks, monitor weekly with dose escalations and tapers
Scroll down to monitoring

Monitoring

- Pain reduction, Functional outcomes, Pain coping. Click here for the Brief Pain Inventory [5]
- Aberrant opioid use
- Medication related side effects

Worsening symptoms

- Assess patient compliance and /or comprehension gaps
- ***REFER*** to pain specialist, chronic pain clinic, rheumatologist or orthopedic surgery as required. (Consider imaging when referring to rheumatology/orthopedic surgery)[1]
- May ***REFER*** to palliative pain clinic depending on institutional policy.

No change in symptoms

- Review exercise/ activity and avoid overuse. Schedule frequent breaks and recovery positions.
- Review individualized goals, medication regimen, and consider alternative therapeutic drug/drug class
- May ***REFER*** to outpatient rehabilitation, pain specialist or chronic pain clinic
- Consider imaging if failure to respond to evidence-based management over a 12-week period[1]

Improvement in symptoms

- Reassess medication regimen as needed
- Engage in physical and self management modalities
- Reinforce exercise (as appropriate)
- Gradually increase exercise to achieve individualized goals

1. Osteoarthritis tool: Canada: The Arthritis Alliance of Canada, the Centre for Effective Practice, the College of Family Physicians of Canada. https://cep.health/media/uploaded/CEP_OATool_2017.pdf. Published 2017. Accessed January 31 2024
2. Ontario renal network (ORN). Pain resource. https://www.ontariorenalnetwork.ca/sites/renalnetwork/files/assets/PainResource_0.pdf. Published 2019. Accessed January 31 2024
3. Project ECHO at University Health Network. Resources. Chronic Pain. Brief pain inventory, https://uhn.echoontario.ca/Resources/Chronic-Pain. Accessed January 31 2024
4. Adams LM, Turk DC. Psychosocial factors and central sensitivity syndromes. *Curr Rheumatol Rev*. 2015; 11: 96–108
5. Project ECHO at University Health Network, ECHO psychosocial screening interview guide, https://uhn.echoontario.ca/Resources/Psychosocial-Resources. Accessed January 31 2024
6. BounceBack Ontario – Canadian Mental Health Association, Ontario, https://bouncebackontario.ca/. Accessed January 31 2024
7. Toronto Academic Pain Medicine Institute (TAPMI), https://tapmipain.ca/healthcare-practitioner/. Accessed January 31 2024
8. Murphy L, Ng K, Isaac P, et al. The Role of the Pharmacist in the Care of Patients with Chronic Pain. *Integr Pharm Res Pract*. 2021; 10: 33–41
9. Arthritis Society of Canada. Osteoarthritis Self-Management - Exercise, Diet, Pain management, https://arthritis.ca/about-arthritis/arthritis-types-(a-z)/types/osteoarthritis/osteoarthritis-self-management. Accessed January 31 2024
10. Dr. Andrea Furlan - YouTube, https://www.youtube.com/c/DrAndreaFurlan. Accessed January 31 2024
11. Calm, https://www.calm.com/. Accessed January 31 2024
12. Lagunju O, Bahl S, Staff M, et al. Mindful, https://www.mindful.org/. Accessed January 31 2024
13. Wilbanks J. Healthy Sleep, https://sleepeducation.org/healthy-sleep/. Accessed January 31 2024.
14. BC Renal agency. BCPRA Guidelines and Drug Choices for Chronic Pain in Dialysis Patients, http://www.bcrenal.ca/resource-gallery/Documents/Guidelines%20and%20Drug%20Choices%20for%20Chronic%20Pain%20in%20Dialysis%20Patients.pdf. Published 2017. Accessed January 31 2024
15. Health Quality Ontario. Quality Standards. Osteoarthritis. https://www.hqontario.ca/Portals/0/documents/evidence/quality-standards/qs-osteoarthritis-clinician-guide-en.pdf. Published 2018. Accessed January 31 2024
16. National Institute for Health and Care Excellence (NICE). Guidance. Osteoarthritis: care and management. https://www.nice.org.uk/guidance/cg177. Published 2022. Accessed January 31 2024
17. Leopoldino AO, Machado GC, Ferreira PH, et al. Paracetamol versus placebo for knee and hip osteoarthritis. *Cochrane Database Syst Rev*. 2019; 2: CD013273
18. da Costa BR, Reichenbach S, Keller N, et al. Effectiveness of non-steroidal anti-inflammatory drugs for the treatment of pain in knee and hip osteoarthritis: a network meta-analysis. *Lancet*. 2017; 390: e21–e33.

19. MacIntyre IM, Turtle EJ, Farrah TE, et al. Regular Acetaminophen Use and Blood Pressure in People With Hypertension: The PATH-BP Trial. *Circulation*. 2022; 145: 416–423.
20. Hughes GJ, Patel PN, Saxena N. Effect of acetaminophen on international normalized ratio in patients receiving warfarin therapy. *Pharmacotherapy*. 2011; 31: 591–597.
21. Murphy L, Babaei-Rad R, Buna D, et al. Guidance on opioid tapering in the context of chronic pain: Evidence, practical advice and frequently asked questions. *Can Pharm J (Ott)*. 2018; 151: 114–120
22. CDC. Guidelines for prescribing opioids for chronic pain. Pocket Guide: Tapering opioids for chronic pain. https://www.cdc.gov/drugoverdose/pdf/clinical_pocket_guide_tapering-a.pdf. Accessed January 31 2024
23. Ivers N, Dhalla IA, Allan GM. Opioids for osteoarthritis pain: benefits and risks. *Can Fam Physician*. 2012; 58: e708
24. Dowell D et al. CDC Clinical Practice Guideline for Prescribing Opioids for Pain — United States, 2022. MMWR Recomm Rep 2022;71(No. RR-3):1–95.
25. Ostelo RWJG, Deyo RA, Stratford P, et al. Interpreting change scores for pain and functional status in low back pain: towards international consensus regarding minimal important change. *Spine (Phila Pa 1976)*. 2008; 33: 90–94
26. Krebs EE, Lorenz KA, Bair MJ, et al. Development and Initial Validation of the PEG, a Three-item Scale Assessing Pain Intensity and Interference. *J Gen Intern Med.* 2009; 24: 733–738.
27. CDC. Guidelines for prescribing opioids for chronic pain, https://www.cdc.gov/drugoverdose/pdf/prescribing/Guidelines_factsheet-a.pdf. Accessed January 31 2024
28. Ontario College of Pharmacists (OCP). Resources on Naloxone and How to Obtain Naloxone Kits, https://www.ocpinfo.com/naloxone-resources/. Published 2017. Accessed January 31 2024
29. Mosca L. Machealth. Opioid conversion table - Safer opioid prescribing strategies, https://machealth.ca/search?q=conversion%20table. Accessed January 31 2024
30. Centre for Effective Practice.Toronto. Opioid Manager. https://cep.health/media/uploaded/CEP_Opioid_Manager_2017.pdf. Published 2017. Accessed January 31 2024
31. Busse JW, Craigie S, Juurlink DN, et al. Guideline for opioid therapy and chronic noncancer pain. *CMAJ.* 2017; 189: E659–E666.
32. Johnson RE, Fudala PJ, Payne R. Buprenorphine: considerations for pain management. *J Pain Symptom Manage*. 2005; 29: 297–326
33. Warner NS, Warner MA, Cunningham JL, et al. A Practical Approach for the Management of the Mixed Opioid Agonist-Antagonist Buprenorphine During Acute Pain and Surgery. *Mayo Clin Proc.* 2020; 95: 1253–1267
34. Powell VD, Rosenberg JM, Yaganti A, et al. Evaluation of Buprenorphine Rotation in Patients Receiving Long-term Opioids for Chronic Pain. *JAMA.* 2021; 4: e2124152
35. Suboxone monograph, https://pdf.hres.ca/dpd_pm/00063411.PDF. Published 2020. Accessed January 31 2024
36. Tobin DG, Lockwood MB, Kimmel PL, et al. Opioids for chronic pain management in patients with dialysis-dependent kidney failure. *Nat Rev Nephrol* 2022; 18: 113–128.

37. Steenhof N, Ng K. Buprenorphine-naloxone in chronic pain: Overcoming stigma for safer opioid management. *Can Pharm J (Ott)*. 2023; 157: 7–9.
38. Butrans monograph, https://pdf.hres.ca/dpd_pm/00035932.PDF. Accessed January 31 2024
39. Paramanandam G, Prommer E, Schwenke DC. Adverse effects in hospice patients with chronic kidney disease receiving hydromorphone. *J Palliat Med*. 2011; 14: 1029–1033.
40. Vondracek SF, Teitelbaum I, Kiser TH. Principles of Kidney Pharmacotherapy for the Nephrologist: Core Curriculum 2021. *Am J Kidney Dis.* 2021; 78: 442–458.
41. College of Physicians and Surgeons of British Columbia. Guidelines Methadone for analgesia, https://www.cpsbc.ca/files/pdf/DP-Methadone-for-Analgesia-Guidelines.pdf. Published 2022. Accessed January 31 2024
